# Modeling Co-Infection by *Streptococcus suis* and *Haemophilus parasuis* Reveals Influences on Biofilm Formation and Host Response

**DOI:** 10.3390/ani13091511

**Published:** 2023-04-29

**Authors:** Mengxia Gao, Jing Zuo, Yamin Shen, Shuo Yuan, Shuji Gao, Yuxin Wang, Yang Wang, Li Yi

**Affiliations:** 1 College of Animal Science and Technology, Henan University of Science and Technology, Luoyang 471000, China; 2 Henan Provincial Engineering Research Center for Detection and Prevention and Control of Emerging Infectious Diseases in Livestock and Poultry, Luoyang 471000, China; 3 College of Life Science, Luoyang Normal University, Luoyang 471934, China

**Keywords:** *Streptococcus suis*, *Haemophilus parasuis*, biofilm, mixed biofilm, mixed infection, bacterial interference

## Abstract

**Simple Summary:**

Clinically, *Streptococcus suis* and *Haemophilus parasuis* often co-occur or mix with each other, causing great harm to the pig industry. Thus, we established a mixed infection model in vitro and a co-infected mice model. We found that the co-existence of *S. suis* and *H. parasuis* can interfere with each other. There was competition between *S. suis* and *H. parasuis* in co-culture. Compared to single cultures, co-cultures showed enhanced biofilm formation, changes in virulence genes, and increased resistance to drugs. The number of bacteria in the co-infected mice increased and the inflammatory response changed. Ultimately, the study elucidated the interaction between *S. suis and H. parasuis*. This provides new ideas for the prevention and treatment of porcine respiratory disease syndrome caused by bacteria.

**Abstract:**

*Streptococcus suis* (*S. suis*) and *Haemophilus parasuis* (*H. parasuis*) are two primary pathogens currently affecting the porcine industry. They often cause encephalitis and arthritis. They also frequently co-infect in clinical settings. In the current study, we identified significant correlations between *S. suis* and *H. parasuis*. The results from CI versus RIR suggested that *S. suis* and *H. parasuis* were competitive in general. Compared to mono-species biofilm, the biomass, bio-volume, and thickness of mixed-species biofilms were significantly higher, which was confirmed using crystal violet staining, confocal laser scanning microscopy, and scanning electron microscopy. Compared to mono-species biofilm, the viable bacteria in the mixed-species biofilms were significantly lower, which was confirmed using the enumeration of colony-forming units (CFU cm^−2^). The susceptibility of antibiotics in the co-culture decreased in the planktonic state. In contrast, biofilm state bacteria are significantly more difficult to eradicate with antibiotics than in a planktonic state. Whether in planktonic or biofilm state, the expression of virulence genes of *S. suis* and *H. parasuis* in mixed culture was very different from that in single culture. Subsequently, by establishing a mixed infection model in mice, we found that the colonization of the two pathogens in organs increased after mixed infection, and altered the host’s inflammatory response. In summary, our results indicate that *S. suis* and *H. parasuis* compete when co-cultured in vitro. Surprisingly, *S. suis* and *H. parasuis* synergistically increased colonization capacity after co-infection in vivo. This study elucidated the interaction between *S. suis* and *H. parasuis* during single infections and co-infections. Future studies on bacterial disease control and antibiotic treatment should consider the interaction of mixed species.

## 1. Introduction

*Streptococcus suis* (*S. suis*) is a commensal bacterium of the upper respiratory tract of pigs and an important zoonotic pathogen that can cause a variety of diseases such as sepsis, arthritis, meningitis, and endocarditis, resulting in huge economic losses [1]. Serotypes have been identified by capsular polysaccharide, and *S. suis* serotype 2 (*S. suis* 2) is the most virulent and most prevalent strain [2]. Studies from our laboratory have demonstrated that *S. suis* 2 has a strong biofilm formation ability, and the biofilm formation ability of the *S. suis* 2 virulent strain is stronger than that of the *S. suis* 2 avirulent strain [3]. Meanwhile, Meng et al., demonstrated that *S. suis* biofilm formation can reduce sensitivity to many antibiotics [4]. Lee, K.W. et al., demonstrated that mixed-species biofilms are more resistant to adverse environments than single-species biofilms [5].

*Haemophilus parasuis* (*H. parasuis*) is a symbiotic Gram-negative bacterial pathogen in the upper respiratory tract of pigs [6]. It is the causative agent of Glaser’s disease, a systemic disease characterized by polyarthritis, fibrinous polyserositis and meningitis [7]. In total, 15 serovars of *H. parasuis* have been identified, with serovars 4 and 5 being the most prevalent worldwide. *H. parasuis* plays an important role during infection after forming a biofilm [8]. Under normal circumstances, *H. parasuis* does not cause disease, but in the case of stress factors or disease infections, it can invade the body and cause serious systemic diseases [9]. *H. parasuis* is an opportunistic pathogen that often co-infected the host with other pathogens. For example, pigs infected with Porcine Reproductive and Respiratory Syndrome Virus (PRRSV) are prone to secondary infections with *H. parasuis*, which induces a strong inflammatory response [10].

Since both *S. suis* and *H. parasuis* are present in the upper respiratory tract and both are the main pathogens of the Porcine Respiratory Disease Complex (PRDC), thus they interact when infecting the host [11]. Mathieu-Denoncourt et al., showed no significant interaction between *S. suis* and *H. parasuis*, but they believe their interaction may be related to biofilm [12]. Bacterial biofilm is a bacterial adherent aggregate encapsulated by extracellular polymer (EPS) [13]. More than 90% of bacteria in nature can form biofilms [14]. Most studies focus on biofilms of a single species, whereas mixed biofilms can more closely mimic the natural disease. The interaction between bacteria can be roughly divided into competition and association, and it will affect the growth and spatial distribution of the population within the biofilm [15,16]. Manyu Jin et al., developed a TaqMan real-time PCR assay that detected *S. suis* and *H. parasuis*, which exhibited efficient identification in mixed biofilms [17]. Reddinger, R.M. et al., showed that *Streptococcus pneumoniae* inhibits *Staphylococcus aureus* biofilm dispersion after *S. aureus* and *S. pneumoniae* form a bi-species biofilm [18]. Pompilio, A. et al., showed that there is mutual interference between *Stenotrophomonas maltophilia* and *Pseudomonas aeruginosa* in cystic fibrosis lung [19]. Cope, E.K. et al., showed that the interaction between nontypeable *Haemophilus influenzae* and *Streptococcus pneumoniae* affects the physical and chemical mechanisms of virulence gene expression in a mixture of biofilm communities [20]. In fact, interspecies interactions are unavoidable and may affect the dynamics of biofilm formation in each species.

Therefore, we investigated the complex interactions between *S. suis* and *H. parasuis* during in vitro biofilm formation and virulence gene expression. We examined the impact of co-infection on bacterial physiology and pathogenesis by observing the entire infection process in mice models with chronic infection. Specifically, we worked with *S. suis* and *H. parasuis* bacterial strains to better understand how these pathogens interact during biofilm formation and infection. This provides new ideas for the prevention and treatment of porcine respiratory disease syndrome caused by bacteria.

## 2. Materials and Methods

### 2.1. Bacterial Strains and Growth Conditions

*H. parasuis* and *S. suis* from clinically ill pigs were isolated by standard methods. The identified causative bacteria were *H. parasuis* type 5 and *S. suis* type 2. *S. suis* was grown in Tryptic Soy Broth (TSB) or Tryptic Soy Agar (TSA) medium at 37 °C. *H. parasuis* was grown in TSB or TSA supplemented with 10 μg/mL of nicotinamide adenine dinucleotide (NAD) and 5% (*v*/*v*) inactivated bovine serum (T/V/S) at 37 °C. Amoxicillin, gentamicin and enrofloxacin were purchased from the China Veterinary Drug Research Institute (Beijing, China). The stock solution (1280 mg/L) of antibiotics was stored at −20 °C.

### 2.2. Mono-Culture and Co-Culture Planktonic Growth Assays In Vitro

Co-culture (3 mL) were inoculated by pure cultures of *S. suis* or *H. parasuis* grown to exponential phase (OD600, 0.5–0.6), diluted to OD600 = 0.05, and mix in a 1:1 ratio in TSB with 10 mg/mL NAD and 5% bovine serum. At different growth stages (0 h, 2 h, 4 h, 6 h, 8 h, 10 h, 12 h and 24 h), samples were serially diluted in sterile phosphate-buffered saline (PBS), plated on TSA and TSA supplemented with 10 mg/mL NAD and 5% bovine serum. In TSA without NAD, *H. parasuis* does not grow. After the plate was incubated at 37 °C for 24 h, the CFU number was recorded. All growth cultures were performed in triplicate.

### 2.3. Establishment of Single and Mixed Biofilms In Vitro

Briefly, pure cultures of *S. suis* or *H. parasuis* that grew to the exponential phase (OD600, 0.5–0.6) were inoculated individually or in a ratio of 1:1 into fresh TSB with 5% bovine serum and 10 mg/mL NAD. Subsequently, the inoculated samples were then individually transferred to 96-well polystyrene, flat bottom, tissue culture-treated microtiter. Add 200 μL of each diluted sample to the sample wells. It was then incubated at 37 °C.

### 2.4. Measurement of Biofilm Biomass

The biomass of the biofilm was determined according to the method previously described [21]. To mimic chronic infection, the medium was changed daily during biofilm formation. The biofilm in each well was washed 3 times with sterile phosphate-buffered saline (PBS, pH 7.0) at each time point (12 h, 24 h, 48 h, 72 h). After drying, it was fixed with 95% methanol for 20 min. Then it was rinsed one time with sterile saline. After drying, it was stained with 0.1% (*w*/*v*) crystal violet for 10 min. It was then rinsed with distilled water and dried. It was then dissolved in 95% ethanol for 5 min. The absorbance was measured at 590 nm using a microplate reader (Infinite 200, Tecan, Switzerland).

After incubation for 24 h to form a biofilm, each well was washed three times with PBS. 100 μL of PBS was added to each well and then sonicated for 5 min using an ultrasonic bath. Serial dilutions were made and plated onto TSA plates with 5% bovine serum and 10 mg/mL NAD to determine the CFU present in the biofilm. The bacteria were counted on the plates as described above.

### 2.5. Observation of Biofilms by Confocal Laser Scanning Microscopy

Mono-culture of *S. suis* or *H. parasuis* and co-culture of a mixture (1:1 ratio) grown in TSB supplemented with 5% bovine serum and 10 mg/mL NAD, were inoculated into 24-well polystyrene tissue culture-treated microtiter plates. The plates were then incubated at 37 °C for 24 h. After washing the plates thrice with PBS, the planktonic cells were removed. After drying at room temperature, the biofilm was labelled with SYTO 9 by the instruction manual from LIVE/DEAD BIOFILM (ABI L10316, Invitrogen, Waltham, MA, USA). Confocal Laser Scanning Microscope (CLSM), Carl Zeiss LSM800, Jena, Germany, was used to analyze the samples.

### 2.6. Observation of Biofilms by Scanning Electron Microscope

Bacterial suspensions of *S. suis*, *H. parasuis* and a combination of *S. suis* and *H. parasuis* in a ratio of 1:1 grown in TSB supplemented with 5% bovine serum and 10 mg/mL NAD, were inoculated into 24-well polystyrene, flat bottom, tissue culture-treated microtiter, then placed in sterile coverslips of appropriate size and incubated at 37 °C for 24 h. Coverslips were washed three times with PBS and fixed with 2.5% glutaraldehyde for 24 h. Then washed three times with PBS for 10 min each. The samples were dehydrated in series of 30%, 50%, 70%, 80%, 90%, and 100% (twice) ethanol for 15 min each. The dehydrated samples were freeze-dried and platinum-coated with an IB-5 ion coater before being examined using a JSM-7800F ultra-high-resolution thermal field emission scanning electron microscope (Japan Electronics, Tokyo, Japan). All SEM images were magnified 5000 times. The images were acquired for three independent copies.

### 2.7. Analysis of Gene Expression in Mixed Planktonic and Biofilm Cells

The virulence gene expression of *S. suis* was measured in combination with *H. parasuis* using relation PCR(RT-PCR) Different combinations of bacterial suspensions (*S. suis*, *H. parasuis* and 1:1 combination) were grown to logarithmic growth phase, according to the method described previously. Following the abovementioned culturing biofilm, it was incubated at 37 °C for 24 h. Then the biofilm was washed with PBS two to three times and sonicated for ten minutes. RNA was extracted using TRIzon (CoWin Biosciences Co., Ltd., Beijing, China) according to the manufacturer’s instructions. Purity was checked by a NanoDrop-2000 spectrophotometer (Thermo Scientific Italia, Milan, Italy). RNA samples were reverse transcribed into cDNA using Prime Script RT-PCR kit (Takara). Gene expression was assessed using SYBR green (Applied Biosystems, Waltham, MA, USA) RT-PCR assay. The gene expression level (arbitrary units) was normalized using 16sRNA as an internal reference. The primer sequences are listed in Table A1, and gene quantification analysis was performed using the 2^−ΔΔCt^ method.

### 2.8. Antibiotic Susceptibility of Planktonic and Biofilm Cultures

The MIC and MBC of the antibiotic were evaluated using a Micro broth dilution method from the Clinical and Laboratory Standards Association standard [22]. Briefly, serial dilutions of antibiotics were prepared in TSB with 5% bovine serum and 10 mg/mL NAD and 100 µL of dilution was added to 96-well microplates (Corning/Costar, NY, USA). Overnight cultures of *S. suis* and *A. pleuropneumoniae* were diluted at 1:100 with TSB contained in 5% bovine serum and 10 mg/mL NAD, and 100 µL of *S. suis*, *H. parasuis*, or combination in 1:1 was added to 96-well plates and incubated at 37◦C for 24 h. The microplates were statically incubated at 37 °C for 24 h. The minimum inhibitory concentration (MIC) values were determined by reading the visual observation of the turbidity. MIC is defined as the lowest concentration of test reagent that completely inhibits visible growth in TSB with 5% bovine serum and 10 mg/mL NAD. MBC were determined by inoculating 2 µL from each well with no visible bacterial growth in TSA plate with 5% bovine serum and 10 mg/mL NAD. The plates were then incubated at 37 °C for 24 h. MBC is defined as the lowest concentration of test reagent that kills 99.9% of the test bacteria after inoculation onto the TSA plate with 5% bovine serum and 10 mg/mL NAD.

Biofilm MIC and MBC measurements were performed as previously described [23]. Different combinations of bacterial suspensions were added to 96-well plates to culture biofilms as described above. After incubation for 24 h at 37 °C, the wells were washed 2–3 times with PBS. Serial dilutions of antibiotics were prepared in TSB with 5% bovine serum and 10 mg/mL NAD, then added to the washed wells and incubated for an additional 24 h. The MIC value was determined by visual observation of the turbidity gradient. Bacteria were counted by CFU count to establish a minimum bactericidal concentration (MBC).

### 2.9. Establishment of Mixed Infection Mice Model

To evaluate the influence of *S. suis* and *H. parasuis* virulence in co-infection, a model of mixed infection with *S. suis* and *H. parasuis* was established. A mixed infection mice model was performed according to a protocol previously published by our group [24].

### 2.10. Determination of Live Bacteria in Organs

To determine the distribution of *S. suis* and *H. parasuis* in different tissues of mice with single infection or co-infection, we divided the experiment into the *S. suis* infection, *H. parasuis* infection, and co-infection (1:1) groups. *S. suis* and *H. parasuis* were cultured overnight at 37 °C under the speed of 190 rpm and diluted serially with sterile PBS to a concentration of 5 × 10^6^ CFU/mL. The diluted bacterial suspension along with PBS were intraperitoneally injected into female BALB/c mice having specific pathogen-free (SPF) into 3 groups (5 mice per group, 4 weeks old). Five mice in each group were euthanized 24 h post-infection. The heart, liver, spleen, lung, brain and trachea were removed and homogenized in 1 mL PBS with 1 mm-diameter zirconia/silica beads, serially diluted in PBS and plated as described above to determine the number of bacterial colonies. Colonies were counted and expressed in colony-forming units CFU/mL.

### 2.11. Inflammatory Factor Expression Assay

The expression of inflammatory factors in the spleen of mice when *S. suis* and *H. parasuis* coexisted were determined. The experiment was divided into the *S. suis* infection, *H. parasuis* infection, and co-infection (1:1) groups. *S. suis* and *H. parasuis* were cultured overnight at 37 °C and then diluted with sterile PBS to a concentration of 5 × 10^6^ CFU/mL. The diluted bacterial suspension and PBS were injected into the peritoneum of specific pathogen-free (SPF) female BALB/c mice (4 weeks old, 3 mice per group). Three mice in each group were euthanized 24 h after infection. The total RNA of each lung sample was extracted using TRIzon (CoWin Biosciences Co., Ltd., Beijing, China) according to the manufacturer’s instructions. The purity was checked by a NanoDrop-2000 spectrophotometer (Thermo Scientific Italia, Milan, Italy). RNA samples were reverse transcribed into cDNA using Prime Script RT-PCR kit (Takara, Shiga, Japan). Gene expression was assessed using a SYBR green (Applied Biosystems) RT-PCR assay.

### 2.12. Statistical Analysis and Interpretative Criteria

GraphPad Prism v7 software is used to analyze the data. One-way analysis of variance (ANOVA) was used to analyze planktonic growth and biofilm formation, and RT-PCR results were used for two-way ANOVA. For in vivo infection experiments, log-rank tests were used to analyze survival data. Values of *p* < 0.05 are considered significant, and all data are expressed as mean ± SD. ** *p* < 0.01; * *p* < 0.05.

In co-culture, the Competitive Index (CI) was defined as the *S. suis*/*H. parasuis* ratio within the output sample divided by the corresponding ratio in the inoculum (input): CI = (*S. suis*/*H. parasuis*) output/(*S. suis*/*H. parasuis*) input, where output and input samples were assessed after plating onto selective media serial dilutions of the sample taken at fixed times or the inoculum (t = 0), respectively [25]. For statistical analyses, CI values were first subjected to a Log transformation for normal distribution, then interpreted as follows: a CI value equal to 0 indicates equal competition of the two species; a positive CI value indicates a competitive advantage for *S. suis*; a negative CI value indicates a competitive advantage for *H. parasuis.* Similar to CI, the Relative Increase Ratio (RIR) was calculated based on the growth results obtained from monocultures of each strain [25]. RIR was calculated as the CFU ratio between *S. suis* and *H. parasuis* after growth within individual infections, divided by the CFU ratio between the strains in their respective initial inocula, and was represented side by side with the corresponding CI for comparison. Comparison of CI and RIR for a given experiment using unpaired Student’s *t*-test, significant differences suggest meaningful competition between species.

## 3. Results

### 3.1. Competition between S. suis and H. parasuis during Co-Culture In Vitro

**Figure 1:**
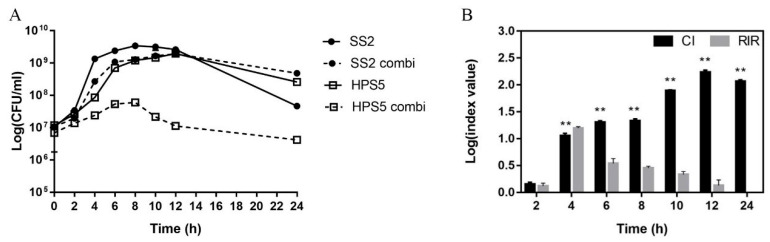
Growth curves and competition indices of pure cultures and co-cultures of *S. suis* and *H. parasuis*. (**A**) Growth curves of *S. suis* and *H. parasuis* strains in pure culture (SS2, HPS5) and in co-culture (*SS*2 combi, *HPS*5 combi). (**B**) Competitive index (CI; black bars) and Relative Increase Ratio (RIR; gray bars) were calculated from single and dual planktonic cultures of *S. suis* and *H. parasuis*. The results are shown as mean ± SD (*n* = 3). ** *p* < 0.01, CI vs. RIR, unpaired *t*-test.

The growth kinetics of *S. suis* and *H. parasuis* in the single or mixed cultures were evaluated by a 24-h colony count (Figure 1A). Both *S. suis* and *H. parasuis* in the co-cultures during both log and stationary phases were inhibited compared to the single culture.

To further evaluate the meaning of the differences observed in the single culture and the co-culture, CI and RIR were calculated and compared (Figure 1B). The CI of *S. suis* and *H. parasuis* was significantly different from the corresponding RIR values between 4 h- and 24 h-incubation and was greater than 0 after Log, indicating that *S. suis* is the dominant bacteria.

### 3.2. Formation of Mixed Biofilms of S. suis and H. parasuis In Vitro

#### 3.2.1. Single or Mixed Biofilms were Visualized by CLSM

**Figure 2:**
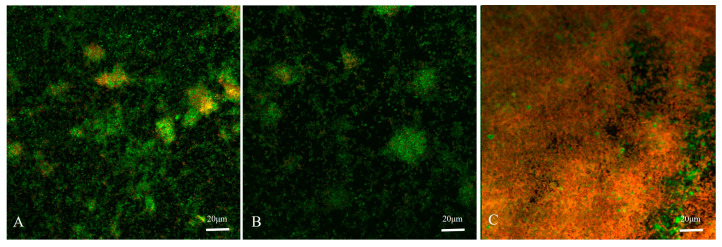
The figure shows the orthogonal views of CLSM images. (**A**) Biofilm formed by *S. suis*; (**B**) Biofilm formed by *H. parasuis*; (**C**) Mixed biofilms formed by *S. suis* and *H. parasuis*. CLSM uses a Carl Zeiss LSM800 confocal scanning system 40× objective lens. LIVE/DEAD BIOFILM staining was used: live cells (with intact cell membranes) stain green and dead or dying cells (with compromised cell membranes) stain red.

The biofilm formed by *S. suis* is lumpy (Figure 2A). The biofilm formed by *H. parasuis* is thinner and more dispersed (Figure 2B). Mixed biofilms formed by *S. suis* and *H. parasuis* are dense and flake (Figure 2C). Furthermore, the viable counts of *S. suis* and *H. parasuis* were more significantly reduced in the co-culture than in the mono-culture.

#### 3.2.2. 3D images of Single and Mixed Biofilms Visualized by Scanning Electron Microscopy

**Figure 3:**
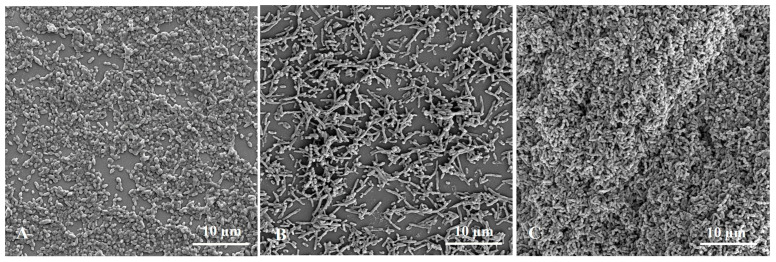
SEM images of *S. suis* and *H. parasuis* single or Mixed biofilms. (**A**) Representative SEM image of *S. suis* single species biofilms. (**B**) Representative SEM images of *H. parasuis* single species biofilms. (**C**) Representative SEM images of mixed biofilms of *S. suis* and *H. parasuis*.

Single or mixed biofilms were visualized by Scanning Electron Microscope (SEM). The biofilm formed by *S. suis* is lumpy (Figure 3A). The biofilm formed by *H. parasuis* is thinner (Figure 3B). The mixed biofilms formed by *S. suis* and *H. parasuis* are dense and flake, *S. suis* and *H. parasuis* are intertwined and closely linked (Figure 3C).

#### 3.2.3. *S. suis* and *H. parasuis* Were Inhibited When Cultured in Mixed Biofilms

**Figure 4:**
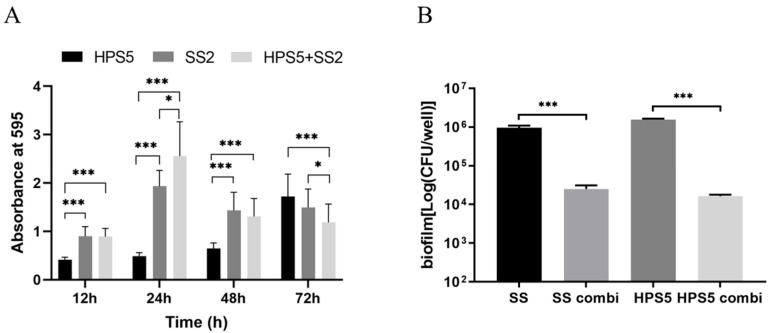
Crystal violet assay and Viable count assay of *S. suis* and *H. parasuis* single or mixed biofilms. (**A**) Crystal violet assay. *S. suis* and *H. parasuis* single (*SS*2, *HPS*5) or mixed biofilms (*SS*2 + *HPS*5) formation after 12 h, 24 h, 48 h, and 72 h incubation were tested. (**B**) Viable count assay. *S. suis* and *H. parasuis* strains were tested in single (SS2, HPS5)or mixed biofilms (*SS*2 combi, *HPS*5 combi). The results are shown as mean ± SD (*n* = 3). * *p* < 0.05, *** *p* < 0.001.

Kinetics of biofilm formation assessed by crystal violet staining (Figure 4A). From 24 h of culture, the biomass of the mixed biofilm is significantly increased compared with the single species biofilm formed by *S. suis* and *H. parasuis*. From 72 h of culture, the biomass of the mixed biofilm is significantly reduced compared with the single-species biofilm formed by *S. suis* and *H. parasuis*.

The interaction of the two bacteria in the biofilm cultured for 24 h was evaluated by viable count (Figure 4B). The number of viable bacteria in *S. suis* and *H. parasuis* biofilms was similar in a single culture. Compared with the single-species biofilms, the viable counts of *S. suis* and *H. parasuis* in the mixed biofilms were significantly reduced and their viable counts were similar.

### 3.3. Mixed Culture of S. suis and H. parasuis Decrease Antibiotic Susceptibility

As shown in Table 1, in the planktonic state, for amoxicillin, MIC increased to 5 μg/mL when co-cultured with *S. suis* and *H. parasuis*. For gentamicin, MIC increased to 20 μg/mL when co-cultured with *S. suis* and *H. parasuis*. In the biofilm state, for gentamicin, the MIC of the mixed biofilms formed by *S. suis* and *H. parasuis* increased to 20 μg/mL. MBC values are higher than 320 μg/mL for both single and mixed biofilms.

### 3.4. H. parasuis Has a Significant Effect on the Virulence of S. suis in In-Vitro Co-Culture

**Figure 5:**
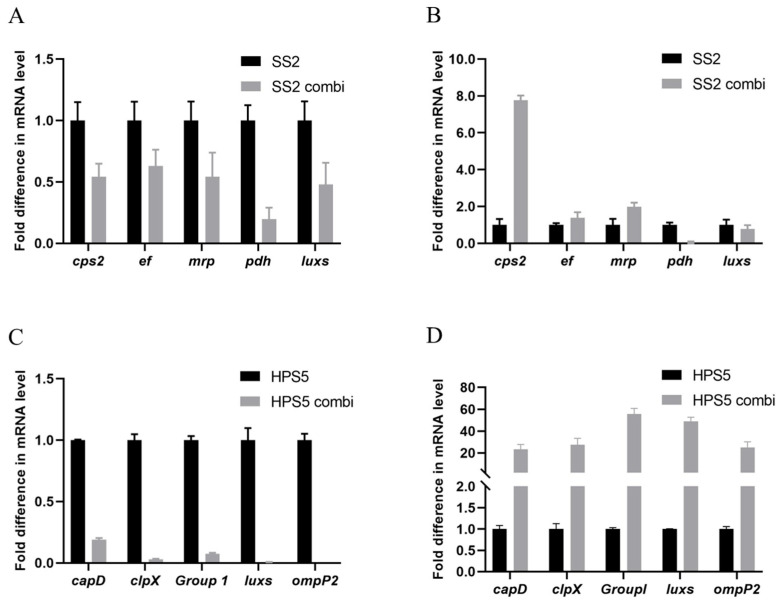
Expression of virulence and quorum sensing-related genes in single and mixed cultures (**A**,**C**) Planktonic state. (**B**,**D**) Biofilm state. The 16SRNA gene was used as a reference. When only *S. suis* or *H. parasuis* is present, the level of *S. suis* or *H. parasuis* gene expression is 100% (*SS*2 or *HPS*5, black bars). When *S. suis* and *H. parasuis* coexisted, the level of *S. suis* or *H. parasuis* gene expression was relative to the level of *S. suis* or *H. parasuis* gene in the presence of only *S. suis* or *H. parasuis* (*SS*2 combi or *HPS*5 combi, gray bars). Data from three independent assays are expressed as mean ± SD.

The expressions of virulence-related genes of *S. suis* and *H. parasuis* were determined by RT-PCR (Table A1). In the planktonic state (Figure 5A,C), the virulence-related genes (*cps2*, *ef*, *mrp*, *pdh*, *luxs*) of *S. suis* were down-regulated (*p* < 0.05), and the virulence-related genes (*capD*, *clpX*, *Group1*, *luxs*, *OmpP2*) of *H. parasuis* were down-regulated (*p* < 0.05). In the mixed biofilms (Figure 5B,D), the *cps2*, *ef*, *mrp* expression of *S. suis* was up-regulated (*p* < 0.05), the expression of *pdh* was down-regulated (*p* < 0.05), and the expression of *luxs* was not significantly different (*p* > 0.05). The virulence-related genes (*capD*, *clpX*, *Group1*, *luxs*, *OmpP2*) of *H. parasuis* were up-regulated (*p* > 0.05). *cps2*, *ef*, *mrp*, *pdh*, *luxs* codifying for capsular polysaccharide, extracellular factor, muramidase-release proteins, Pyruvate dehydrogenase, and S-Ribosylhomocysteinase, respectively. *capD*, *clpX*, *Group1*, *luxs*, *OmpP2* codifying for capsular polysaccharide, casein hydrolytic protease, trimer autotransporter, s-ribohomocysteinase and outer membrane protein respectively.

### 3.5. Coexistence of S. suis and H. parasuis Increase the Bacterial Load in Mice

**Figure 6:**
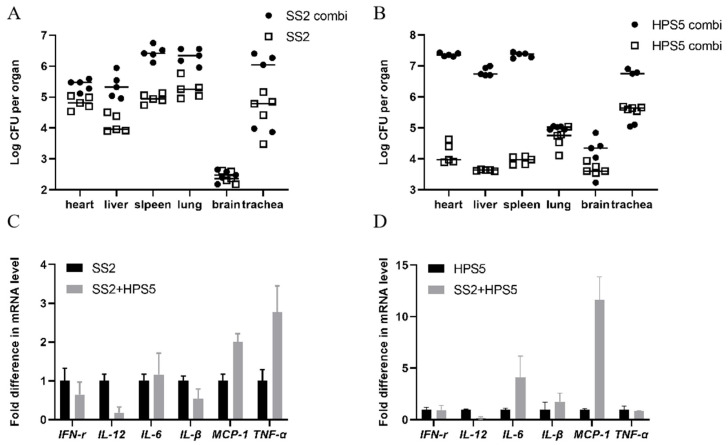
(**A**,**B**) Bacterial counts in different organs. Number of *S. suis* or *H. parasuis* in heart, liver, spleen, lung, brain, and trachea after single infection with *S. suis* or *H. parasuis* (open square, SS2 or HPS5). Number of *S. suis* or *H. parasuis* in heart, liver, spleen, lung, brain and trachea after mixed infection with *S. suis* and *H. parasuis* in mice (closed circles, SS2 or HPS5 combi). The black horizontal line indicates the average value. (**C**,**D**) Expression of inflammatory factor-related genes in mice. The level of gene expression in mice infected with *S. suis* or *H. parasuis* was 100% (SS2 or HPS5, black bars). The level of gene expression in mice infected with *S. suis* and *H. parasuis* was relative to the level of gene expression in mice infected with *S. suis* or *H. parasuis*(SS2 + HPS5, gray bars). Data from three independent determinations are expressed as mean ± SD.

In the mixed infection, the bacterial load of *S. suis* and *H. parasuis* in each organ increased. After collecting the heart, liver, spleen, lung, brain, and trachea of the mice, the distribution of *S. suis* or *H. parasuis* in various organs of the mice was analyzed. As shown in (Figure 6A,B), there was a significant difference in the content of *S. suis* and *H. parasuis* in mice after single infection and mixed infection. The bacteria count of *S. suis* and *H. parasuis* was significantly increased in mice after mixed infection with *S. suis* and *H. parasuis.*

### 3.6. Single and Mixed Infections Affect Inflammatory Factor Expression

As shown in Figure 6C, compared with *S. suis*-infected mice, the mRNA expressions of *IFN-γ*, *IL-12* and *IL-β* were down-regulated, while mRNA expression of *MCP-1*, *TNF-α* and *IL-6* were up-regulated in the co-infected mice. (*p* < 0.05); As shown in Figure 6D, compared with *H. parasuis*-infected mice, the mRNA expressions of *IFN-γ*, *IL-12* and *TNF-α* were down-regulated, while mRNA expression of *MCP-1*, *IL-β* and *IL-6* were up-regulated in the co-infected mice. (*p* < 0.05).

## 4. Discussion

Although the role of biofilms in porcine respiratory infections has garnered increased attention, previous research has primarily focused on single-species biofilms. The research findings highlighted that *S. suis* and *H. parasuis* were the major respiratory tract pathogens responsible for respiratory disease syndrome in pigs. Accordingly, we established mixed biofilm to explore their interactions. Firstly, we used CLSM and SEM to image the biofilm formation and confirm that *S. suis* and *H. parasuis* can form mixed biofilms. It can be seen from CLSM images that the mixed biofilms are denser compared to a single-species biofilm. It can be seen from the CLSM images that the spatial distribution of the mixed biofilms belongs to the “Coaggregation” mode [25]. It cannot be completely determined by imaging that the mixed biofilm becomes stronger or weaker. Therefore, we measured the biomass of the biofilm and the number of viable bacteria under the biofilm by crystal violet staining and colony-forming units (CFUs) respectively. The results showed that the mixed biofilm formed by *S. suis* and *H. parasuis* increased, but the proportion of dead bacteria increased. It is worth noting that the competitiveness of *H. parasuis* in the biofilm state is enhanced compared to the planktonic state. Competitive interactions have been shown to exist primarily between phylogenetically and metabolically similar species [26]. Previous work has shown that the mixed biofilm formed by *S. aureus* and *E. coli* was more complex and changed over time [27]. Studies by Kreth et al., have shown that oral *Streptococcus*, haemorrhagic *Streptococci* and Gordon’s *Streptococcus* inhibit the growth of other oral bacteria, including *Streptococcus* mutants, by producing hydrogen peroxide (H_2_O_2_) in a mixed biofilm [28]. An et al., suggested that *P. aeruginosa* uses strong athletic ability to “cover” *Agrobacterium tumefaciens* to change the spatial distribution in mixed biofilms, thereby leading the competition in hybrid biofilms [29]. It has not been confirmed that *S. suis* has antibacterial activity against *H. parasuis*, and vice versa. Additionally, the imaging results did not show an “overlay” phenomenon. Consequently, the main factor that reduces the number of viable bacteria in the mixed biofilm formed by *S. suis* and *H. parasuis* is the competition for nutrients. The competitive ability of *H. parasuis* is enhanced in the biofilm state in comparison to the planktonic state. This suggests that the balance of the competition between *S. suis* and *H. parasuis* in a mixed biofilm community has changed. Currently, it has been confirmed that *S. suis* and *H. parasuis* have LuxS/AI-2 quorum sensing systems [30,31]. Recent research indicates that AI-2 plays a significant role in regulating virulence-related genes, pathogenicity, and biofilm formation, the extent of which varies depending on the type of bacterium. “Coaggregation” mode of direct contact between microorganisms in the mixed biofilms can facilitate communication more effectively [32]. It can be inferred that the interaction between *S. suis* and *H. parasuis* in the mixed biofilm is related to the bacterial communication.

It is well known that bacteria in a biofilm state are more difficult to eradicate than bacteria in a planktonic state [33,34]. Compared to biofilms formed by a single species, mixed biofilms can more effectively evade host immune defenses and display reduced antibiotic sensitivity [35]. The study showed that co-cultures of *S. suis* and *H. parasuis* in planktonic states reduced sensitivity to antibiotics. Although the coexistence of *S. suis* and *H. parasuis* caused a reduction in the number of bacteria, the sensitivity to the drug did not increase. In the biofilm state, compared with the single biofilms, the mixed biofilms formed by *S. suis* and *H. parasuis* had no significant difference in antibiotic sensitivity, and only the sensitivity to gentamicin was found to be weakened. It is worth noting that none of the drugs utilized in this study entirely eradicated the formed biofilm. In short, whether in the planktonic state or in the biofilm state, the coexistence of the two substances will change the sensitivity of certain antibiotics.

The interactions between microorganisms are complex and play an important role in the infection process [36]. There is competition and coexistence between them. This study indicates that *S. suis* and *H. parasuis* compete, and the CI and RIR values indicate that *S. suis* has a competitive advantage. Antagonistic mechanisms occur in various forms, including one bacterium’s antibacterial effect on another bacterium, chemical signaling that disrupts behavior or physiology, and competition for nutrition and living space. [37]. From the results of a number of viable bacteria under biofilm, *S. suis* has no antibacterial activity against *H. parasuis*, and vice versa. To date, there have been no reports of *S. suis* or *H. parasuis* producing chemical signals that interfere with another bacterium.

Research has demonstrated that virulence and gene expression of bacterial pathogens can be modulated by the presence of other bacterial species [38]. Therefore, we have compared the transcription levels of virulence factors of *S. suis* and *H. parasuis* during the mixed infection in both the planktonic and the biofilm states. Quantitative PCR analysis revealed that the presence of *H. parasuis* reduced the virulence of *S. suis* in the planktonic state and changed the virulence of *S. suis* in the biofilm state. The mixed biofilm significantly increased the expression of *S. suis* capsular polysaccharide (CPS2) and muramidase-release proteins (MRP). The presence of *S. suis* reduced the virulence of *H. parasuis* in the planktonic state and changed the virulence of *H. parasuis* in the biofilm state. The gene expression caused by the interaction between *S. suis* and *H. parasuis* in a biofilm state is more complicated.

Mice models of *S. suis* infection are often established through intraperitoneal [39], intranasal [40], intravenous [41], and intracisternal [42] routes of infection. Mice models of *H. parasuis* infection are often established through intraperitoneal routes of infection [43]. However, intraperitoneal injection models are often used in virulence studies [44,45,46,47]. To further evaluate whether the virulence of co-infection changes, we established a mixed infection mice model of *S. suis* and *H. parasuis* through intraperitoneal routes of infection. Through organ infection experiments and the determination of inflammatory factor transcription levels, we confirmed that the virulence of *S. suis* or *H. parasuis* was enhanced in the mixed infection. The presence of both *S. suis* and *H. parasuis* in mice was found to enhance the colonization of these bacteria in the heart, liver, spleen, lungs and trachea. Co-infection of mice with *S. suis* and *H. parasuis* was found to alter the inflammatory response. In summary, our research shows that the colonization of mice organs by *S. suis* and *H. parasuis* is increased in the presence of co-infection.

## 5. Conclusions

**Figure 7:**
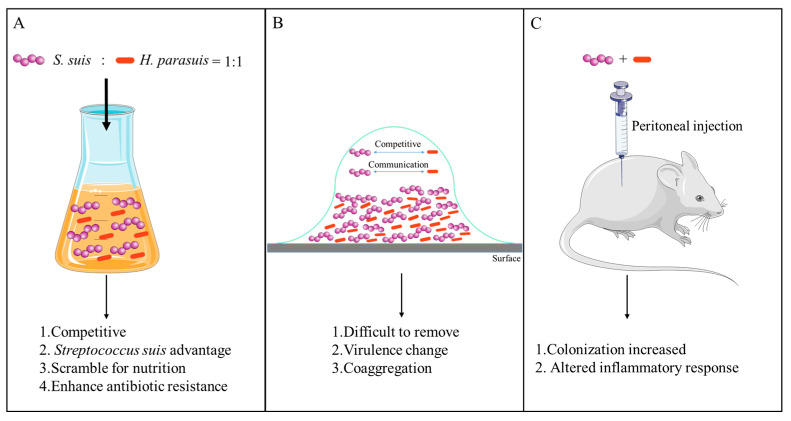
(**A**) Planktonic mixed culture. (**B**) Mixed biofilm model. (**C**) Mice mixed infection model.

The study confirmed a competitive and co-existing relationship between *S. suis* and *H. parasuis* in vitro. *S. suis* is more competitive than *H. parasuis* in planktonic co-cultures (Figure 7A). Additionally, planktonic co-culture enhances drug resistance. A coaggregation pattern developed when *S. suis* and *H. parasuis* were co-cultured in the biofilm state. *S. suis* and *H. parasuis* have similar competition, and the expression of virulence genes is changed (Figure 7B). Compared to bacteria in the planktonic state, bacteria in the biofilm state are significantly more resistant to antibiotics and hinder the eradication of these bacteria. At the same time, we established a mixed infection model of *S. suis* and *H. parasuis* in mice, and we found that the colonization of *S. suis* and *H. parasuis* in organs increased after mixed infection (Figure 7C). The co-culture of *S. suis* and *H. parasuis* caused an alteration in the host inflammatory response. Interactions between different bacterial species could be accountable for the escalation in pathogenicity. Our research establishes a basis for future research on the co-infection of *S. suis* and *H. parasuis*.

## Figures and Tables

**Table 1:** The MIC and MBC of mono-cultures and co-cultures of *S. suis* and *H. parasuis* in planktonic and biofilm states.

Bacterial Combinations	MIC (μg/mL) (Planktonic)	MBC (μg/mL) (Planktonic)	MIC (μg/mL) (Biofilm)	MBC (μg/mL) (Biofilm)
Amoxicillin				
SS2	0.3125	2.5	5	>320
HPS5	0.1625	0.625	10	>320
SS2 + HPS5	5	10	10	>320
Gentamicin				
SS2	10	40	5	>320
HPS5	5	10	10	>320
SS2 + HPS5	20	40	20	>320
Enrofloxacin				
SS2	0.3125	0.625	1.25	>320
HPS5	0.1625	0. 625	0.625	>320
SS2 + HPS5	0.625	20	1.25	>320

## Data Availability

All the data generated or analyzed during this study are available from the corresponding author upon reasonable request.

## Appendix A

**Table A1:** Primers used in this study.

Primers	Primers Sequence (5′–3′)	Target Gene
CPS2-F	ATTGGTAGGCACTGTCGTTGGTC	cps2
CPS2-R	AGAACTTAGCATTGTTGCGGTGG	cps2
EF-F	TCCAATCACAGATCCAGATAGCG	ef
EF-R	CTGACCCATTTGGACCATCTAAG	ef
MRP-F	CAAGGAAAGTGAACAGAACGAGC	mrp
MRP-A	TAGTCGTCCAAACCTGAGTAGCG	mrp
PDH-F	CGCGAATTCATGCAACAAATCCGTGAT	pdh
PDH-R	CCCTCGAGGCTAGTCTACAAACACATC	pdh
LUXS-F	ATGAAAAAAGAAGTCACT	luxs
LUXS-R	TTAGATTGGTTTTCTTTC	luxs
16S RNA-F	GTTGCGAACGGGTGAGTAA	16sRNA
16S RNA-R	TCTCAGGTCGGCTATGTATCG	16sRNA
CAPD-F	ATGTTAATGCCATTAATTTATTCATTG	CapD
CAPD-R	TCGAACCGATAGAACCAGCAGCACCAGTC	CapD
CLPX-F	AGAGTGAGGGCGTTGAGT	clpX
CLPX-R	TTCTTGTTTCGGGTGTTT	clpX
GROUP 1-F	TTTAGGTAAAGATAAGCAAGGAAATCC	Group 1
GROUP 1-R	CCACACAAAACCTACCCCTCCTCC	Group 1
LUXS-F	GCATCAGCAAGAGAATGTTCCT	luxs
LUXS-R	ATGTCGTTAATTGGTTCACCTTCA	luxs
OMPP2-F	ATGAAAAAAACACTAGTAGCA	ompP2
OMPP2-R	TTACCATAATACACGTAAACC	ompP2
16S RNA-F	GGCTTCGTCACCCTCTGT	16sRNA
16S RNA-R	GTGATGAGGAAGGGTGGTGT	16sRNA
IL-6-F	CTGCAAGAGACTTCCATCCAG	IL-6
IL-6-R	AGTGGTATAGACAGGTCTGTTGG	IL-6
IL-12-F	GTCCTCAGAAGCTAACCATCTCC	IL-12
IL-12-R	CCAGAGCCTATGACTCCATGTC	IL-12
IL-1β-F	GAAATGCCACCTTTTGACAGTG	IL-1β
IL-1β-R	TGGATGCTCTCATCAGGACAG	IL-1β
MCP-F	GCATCCACGTGTTGGCTCA	MCP-1
MCP-R	CTCCAGCCTACTCATTGGGATC	MCP-1
TNF-α-F	CAGGCGGTGCCTATGTCTC	TNF-α
TNF-α-R	CGATCACCCCGAAGTTCAGTAG	TNF-α
IFN-γ-F	ACAGCAAGGCGAAAAAGGATG	IFN-γ
IFN-γ-R	TGGTGGACCACTCGGATGA	IFN-γ
GAPDH-F	AGGTCGGTGTGAACGGATTTG	gapdh
GAPDH-R	GGGGTCGTTGATGGCAACA	gapdh
